# Thermal ablation for visible cervical HSIL or suspected HSIL: a tissue-conserving alternative to excision in selected patients

**DOI:** 10.3389/fonc.2026.1833596

**Published:** 2026-05-20

**Authors:** Yan Zhou, Yunfeng Zhang, Dongping Rao, Ying Ma, Ruijiao Zhao, Rujia Fan, Yue Wang, Wenxin Zheng

**Affiliations:** 1 Department of Obstetrics and Gynecology, Henan Provincial People’s Hospital, Zhengzhou University People’s Hospital, Henan University People’s Hospital, Zhengzhou, Henan, China; 2 Department of Obstetrics and Gynecology, Zhengzhou University People’s Hospital, Henan Provincial People’s Hospital, Zhengzhou, Henan, China; 3 Department of Obstetrics and Gynecology, Ningbo Women and Infant’s Hospital, Ningbo, Zhejiang, China; 4 Department of Obstetrics and Gynecology, Mianyang Central Hospital, Mianyang, Sichuan, China; 5 Department of Pathology, Henan Provincial People’s Hospital, Zhengzhou University People’s Hospital, Henan University People’s Hospital, Zhengzhou, Henan, China; 6 Department of Pathology, University of Texas Southwestern Medical Center, Dallas, TX, United States; 7 Department of Obstetrics and Gynecology, University of Texas Southwestern Medical Center, Dallas, TX, United States

**Keywords:** cervical squamous intraepithelial lesion, loop electrosurgical excision procedure, SCJ cells, squamous-columnar junction ablation, thermal ablation

## Abstract

**Background:**

This study evaluates the feasibility and clinical outcomes of squamocolumnar junction (SCJ)-targeted thermal ablation in patients with visible cervical lesions diagnosed as high-grade squamous intraepithelial lesions (HSIL) or suspected HSIL, and explores changes in CK7-positive SCJ cells following treatment.

**Methods:**

In this prospective cohort study, 226 patients meeting predefined eligibility criteria between June 2017 and December 2024 were managed with either SCJ-targeted thermal ablation (n = 110) or loop electrosurgical excision procedure (LEEP, n = 116), based on clinical assessment and patient preference. Outcomes included operative parameters, complications, cytological abnormalities, high-risk HPV (hrHPV) clearance, lesion persistence or recurrence, and changes in CK7-positive SCJ cells in a subset of patients.

**Results:**

Baseline clinicopathologic characteristics were broadly comparable between groups, although treatment allocation was non-randomized. SCJ ablation was associated with shorter operative time, minimal intraoperative blood loss, fewer procedure-related complications, and lower treatment cost compared with LEEP (*P* < 0.05). During follow-up, rates of cytological abnormalities and lesion recurrence were low in both groups, with no progression to invasive carcinoma observed. A higher rate of hrHPV positivity was noted in the ablation group at 3 months (36.4% vs. 18.1%, *P* = 0.006), but this difference was not observed at subsequent time points. In a subset analysis, CK7-positive SCJ cells markedly decreased following both treatments, with no residual CK7-positive HSIL or SCJ cells identified post-treatment.

**Conclusion:**

In carefully selected patients with fully visible transformation zones, SCJ-targeted thermal ablation demonstrated favorable procedural outcomes and similar observed short- to mid-term clinical results compared with LEEP in this observational cohort. These findings support its potential as a tissue-conserving treatment option in selected cases. The reduction in CK7-positive SCJ cells suggests a possible biologic basis for SCJ-targeted therapy, although this remains exploratory. Given the non-randomized design and limited event rates, these findings should be interpreted with caution, and further prospective studies are warranted.

## 1 Introduction

Cervical cancer is the fourth most common malignancy and a leading cause of cancer-related deaths among women worldwide (1). In low- and middle-income countries, it remains the second most common cancer and the second leading cause of cancer-related mortality (1). Cervical intraepithelial neoplasia (CIN) is a premalignant lesion of cervical cancer, histologically classified as high-grade and low-grade squamous intraepithelial lesions (HSIL and LSIL), primarily attributed to persistent infection with high-risk human papillomavirus (hrHPV) (2).

The transformation zone (TZ) of the cervix, anatomically defined as the region between the original and newly formed squamocolumnar junction (SCJ), is recognized as the most susceptible site for persistent high-risk human papillomavirus (hrHPV) infections, which can give rise to cervical intraepithelial neoplasia and, eventually, cervical cancer. This area is biologically active and undergoes dynamic epithelial remodeling during a woman’s reproductive years, making it particularly vulnerable to oncogenic HPV integration. Based on the visibility of the SCJ under colposcopic examination, the TZ is classified into three types: TZ-1, where the SCJ is entirely visible on the ectocervix; TZ-2, where the SCJ is partially located within the endocervical canal but becomes completely visible upon manipulation or exposure; and TZ-3, where the SCJ lies partially or entirely within the endocervical canal and cannot be fully visualized. This classification is crucial for guiding diagnostic and therapeutic decisions, especially in the management of cervical intraepithelial lesions (3).

The SCJ is a histologic landmark and a key site for cervical squamous cell carcinoma development. Recent research has identified a small population of CK7-positive residual embryonic stem or progenitor cells within this region, known as SCJ cells, which are highly susceptible to hrHPV infection and may undergo malignant transformation leading to HSIL (4). The progression from hrHPV infection to cervical cancer generally occurs over a decade. Early detection of squamous intraepithelial lesions (SIL) through screening and timely treatment of HSIL significantly reduce cervical cancer incidence.

For the treatment of premalignant cervical lesions, both ablative methods (cervical cryotherapy, laser ablation) and excisional methods (loop electrosurgical excision procedure [LEEP], cold knife conization) can be therapeutic. While excisional procedures allow for histopathological assessment, they also increase the risk of adverse pregnancy outcomes, including miscarriage, preterm birth, and cervical insufficiency, with rates as high as 14% (5, 6). SCJ ablation, which removes SCJ cells while preserving cervical integrity, offers an alternative approach that minimizes these risks. However, to ensure safety, SCJ ablation is only considered when invasive cancer is ruled out, the TZ and lesion are fully visible, HSIL is absent in the endocervical canal, and the lesion remains within a treatable range. Despite its potential benefits, SCJ ablation is underutilized due to limited understanding of the progression of hrHPV-infected SCJ cells.

Despite increasing recognition of the role of SCJ cells in the pathogenesis of cervical neoplasia (4, 7), clinical evidence supporting SCJ-targeted treatment strategies for high-grade squamous intraepithelial lesions (HSIL) remains limited. Most existing studies have focused on excisional approaches such as LEEP or ablative methods without specifically targeting the SCJ (5, 6, 8), and a few have prospectively compared SCJ-directed ablation with standard excisional treatment in well-defined patient populations (9). Furthermore, the relationship between SCJ cell dynamics, as reflected by biomarkers such as CK7, and clinical treatment outcomes has not been well characterized in prospective clinical settings. These gaps limit the ability to translate emerging biologic insights into practical treatment strategies.

In this prospective cohort study, we aimed to evaluate the feasibility and clinical outcomes of SCJ-targeted thermal ablation compared with LEEP in patients with visible HSIL or suspected HSIL, and to explore the potential association between treatment response and changes in CK7-positive SCJ cells.

## 2 Materials and methods

### 2.1 Patient selection

This prospective cohort study included patients presenting with cervical squamous intraepithelial lesions (SIL) or lesions suspected to represent high-grade squamous intraepithelial lesions (HSIL) at the Cervical Diagnosis and Treatment Centers of Henan Provincial People’s Hospital and Ningbo Women and Children’s Hospital between June 2017 and December 2024. Eligibility was based on a combination of cytologic, colposcopic, and histologic assessment consistent with clinical practice for HSIL management.

Inclusion criteria were: age ≤40 years, desire for fertility preservation, abnormal cervical cytology (up to HSIL), positive high-risk HPV testing, histologic diagnosis of HSIL or lesions suspicious for HSIL on biopsy, negative endocervical curettage (ECC), and a fully visible transformation zone (TZ type 1 or 2). Patients with type 3 transformation zones were excluded due to limited visualization.

Exclusion criteria included pregnancy, prior treatment for HSIL, evidence of invasive carcinoma, glandular involvement, significant endocervical extension, bleeding disorders, immunodeficiency, or severe cervical inflammation.

A total of 226 patients were enrolled, including 110 who underwent SCJ-targeted thermal ablation and 116 who received LEEP.

### 2.2 Treatment allocation

Treatment assignment was not randomized. The choice between SCJ-targeted thermal ablation and LEEP was based on a combination of patient preference (particularly regarding desire to preserve cervical integrity), clinician assessment, and predefined eligibility criteria, including lesion visibility, extent, and absence of endocervical or glandular involvement. Both treatment options were discussed with patients prior to decision-making. All procedures were performed by experienced gynecologists trained in both techniques.

### 2.3 Procedures

**Figure 1:**
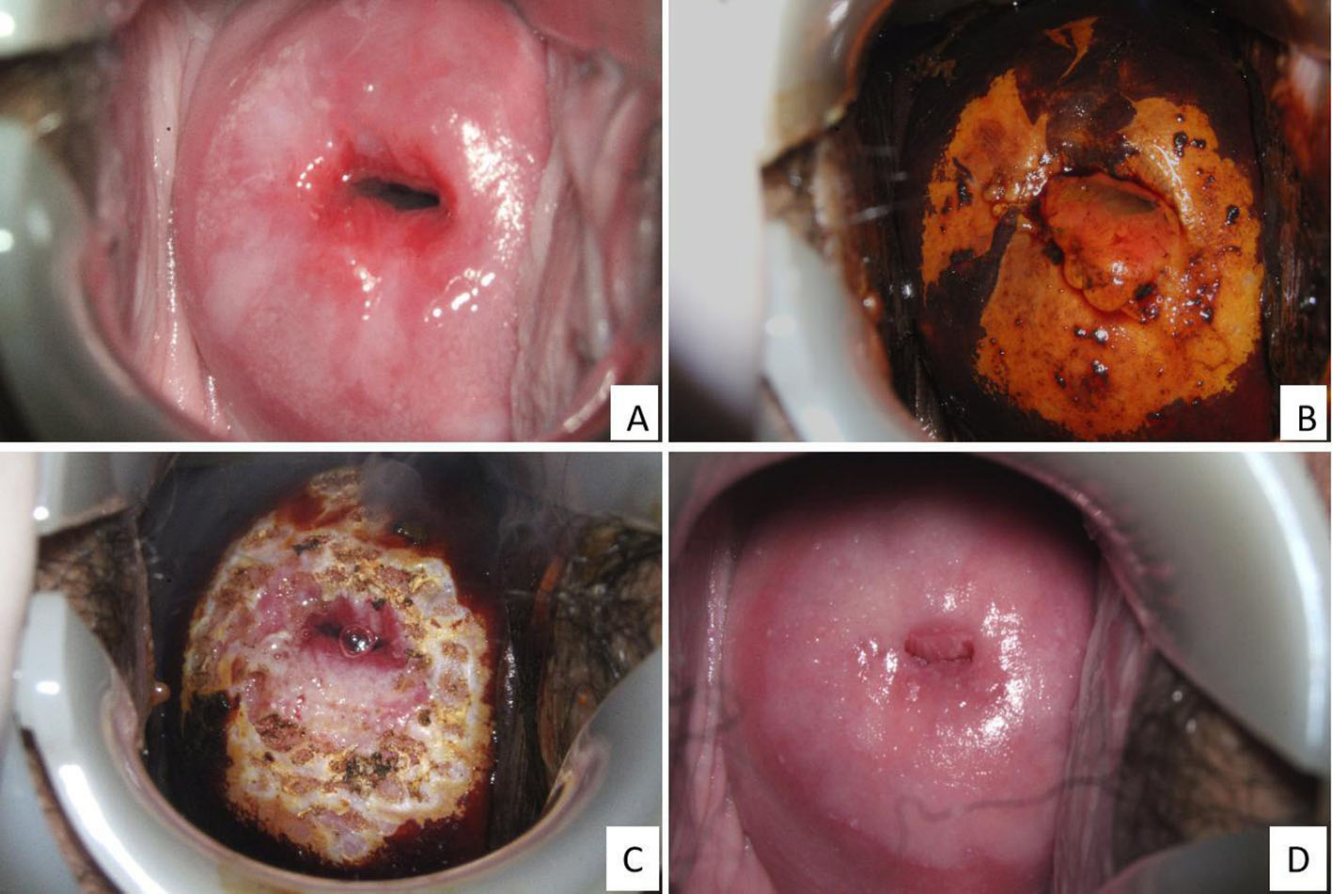
Colposcopic findings before and after electrocautery ablation in a CIN3. Colposcopic images from a 30-year-old woman with a high-grade squamous intraepithelial lesion (HSIL) on Pap smear and positive high-risk HPV testing. **(A)** Acetowhite epithelium indicating HSIL; **(B)** Iodine-negative areas corresponding to abnormal epithelium; **(C)** Immediate post-ablation appearance following electrocautery; and **(D)** Newly formed squamocolumnar junction (SCJ) observed at the three-month follow-up examination.

Prior to SCJ ablation, all patients underwent colposcopic re-evaluation to exclude invasive carcinoma. Cases were excluded from the ablation group if the lesion extended over more than 75% of the cervical surface, involved the endocervical canal, type 3 TZ, or showed evidence of glandular epithelial involvement. SCJ ablation was performed with the patient in the lithotomy position. After routine disinfection, a 5% povidone-iodine solution was applied to the cervix. Lesion margins and the SCJ were identified using colposcopic imaging. Thermal ablation was carried out using an electrocautery probe, extending 5–7 mm beyond the lesion margins. Ablation depth was limited to within 3 mm and continued until the treated tissue appeared coagulated and yellowish-white in color (**Figure 1**). If needed, hemostasis was achieved with gelatin sponges and gauze compression.

LEEP was performed in accordance with the 2011 guidelines of the International Federation for Cervical Pathology and Colposcopy (IFCPC) (10). Patients were placed in the lithotomy position and received local anesthesia. Following cervical exposure and iodine staining to delineate the lesion margins, a high-frequency electrosurgical loop was used to excise the lesion with a 0.3–0.5 cm margin. The depth and extent of excision were tailored based on lesion severity and patient fertility status. Excised specimens were submitted for histopathological analysis. Hemostasis was ensured with electrocautery and hemostatic gauze packing.

### 2.4 HPV detection, cytology, histopathology and immunohistochemistry evaluation

Cervical specimens were obtained from the transformation zone using a specialized cervical brush for both high-risk HPV genotyping and cytological evaluation. HPV testing was conducted with either the Cobas (Roche, Beijing) or the APTIMA HPV16/18/45 Genotype Assay (Hologic Inc., Beijing), a nucleic acid amplification test based on target capture hybridization. Cytological assessment was performed via liquid-based cytology using the ThinPrep 2000 system, and slides were interpreted by experienced cytopathologists. Cervical tissue samples collected before and after treatment were subjected to histopathological evaluation by a gynecologic pathologist (RZ) and reviewed by another senior consultant (WZ) if diagnostic difficulties encountered. Immunohistochemical (IHC) staining for CK7 was then performed on these samples to assess the presence of SCJ cells. CK7 positivity was defined as strong cytoplasmic staining in more than ten contiguous non-columnar epithelial cells. Importantly, CK7-positive cells associated with HSIL were distinguished from those originating from SCJ. In cases where a CIN2 diagnosis was suspected, additional p16 immunostaining was utilized to support the histologic interpretation, with block-positive staining indicative of HSIL.

### 2.5 Follow-up and outcome measures

Patients were followed at 3, 6, 9, and 12 months post-treatment with repeat cytology and high-risk HPV testing. Abnormal findings prompted colposcopic evaluation and biopsy when indicated.

The primary clinical outcomes of interest were lesion persistence or recurrence (including HSIL and LSIL) and high-risk HPV clearance. Secondary outcomes included operative time, intraoperative blood loss, treatment cost, postoperative complications, and cytologic abnormalities during follow-up.

Changes in CK7-positive SCJ cells before and after treatment were evaluated in a subset of patients as an exploratory biomarker analysis.

Extended follow-up beyond one year was available for a subset of patients and is reported separately.

### 2.6 Statistical analysis

Statistical analyses were performed using SPSS 20.0 software. Continuous variables are presented as mean ± standard deviation and compared using Student’s t-test. Categorical variables are presented as counts and percentages and compared using chi-square or Fisher’s exact test where appropriate.

Given the non-randomized design and low event rates, analyses were primarily descriptive and exploratory. No formal non-inferiority or equivalence testing was performed. Multivariable adjustment was not conducted due to limited outcome events. A P-value <0.05 was considered statistically significant.

## 3 Results

### 3.1 Comparison of baseline clinical characteristics between the SCJ ablation and LEEP groups

Baseline clinical characteristics of patients with HSIL in the SCJ ablation and LEEP groups are summarized in **Table 1**. The SCJ ablation group consisted of 110 patients aged 22 to 40 years, with a mean age of 30.6 ± 5.8 years. Colposcopic evaluation classified 60 cases (55.5%) as TZ-1 type and 50 cases (44.5%) as TZ-2 type. Colposcopic diagnosis indicated ≤LSIL in 66 cases (60.0%) and HSIL in 44 cases (40.0%). Among the 44 HSIL cases, lesion size ranged from 0.4 to 2.5 cm², with a mean size of 1.62 cm². Pre-ablation histopathology confirmed ≤LSIL in 62 patients (56.4%) and HSIL in 48 patients (43.6%). The concordance rate between colposcopic and histological diagnoses was 95.5%.

**Table 1:** Baseline clinical and pathological data between SCJ ablation and LEEP groups.

Category	SCJ ablation group (n=110)	LEEP group (n=116)	X²	*P*-value
Age (years)			0.65	0.421
<25	7 (6.4%)	4 (3.4%)		
25-40	103 (93.6%)	112 (96.6%)		
Parity			7.28	0.007
0 pregnancies	46 (41.8%)	22 (18.9%)		
≥1 pregnancy	64 (58.2%)	94 (82.1%)		
TCT Results			0.07	0.966
NILM (Negative)	38 (35.5%)	41 (35.3%)		
ASCUS & LSIL	44 (40.0%)	45 (38.8%)		
ASC-H & HSIL	28 (25.5%)	30 (25.9%)		
High-Risk HPV			0.00	0.970
HPV 16/18/45	103 (93.6%)	107 (92.2%)		
Other High-Risk Types	7 (6.4%)	9 (7.8%)		
Colposcopic Evaluation			0.00	0.939
≤ LSIL	66 (60.0%)	71 (61.2%)		
HSIL	44 (40.0%)	45 (38.8%)		
TZ Classification			0.03	0.856
TZ-1	60 (55.5%)	59 (50.8%)		
TZ-2	50 (44.5%)	57 (49.2%)		
Histological Diagnosis*			0.00	0.955
LSIL	62 (56.4%)	66 (56.9%)		
HSIL	48 (43.6%)	50 (43.1%)		

TCT, ThinPrep Cytology Test; NILM, Negative for Intraepithelial Lesion or Malignancy; ASCUS, Atypical Squamous Cells of Undetermined Significance; LSIL, Low-Grade Squamous Intraepithelial Lesion; ASC-H, Atypical Squamous Cells, Cannot Exclude HSIL; HSIL, High-Grade Squamous Intraepithelial Lesion; HPV, Human Papillomavirus; TZ, Transformation Zone; *Histological diagnosis represents pre-ablation or pre-LEEP findings.

The LEEP group included 116 patients aged 23 to 40 years, with a mean age of 32.6 ± 5.3 years. Of these, 59 cases (50.8%) were classified as TZ-1 and 57 cases (49.2%) as TZ-2. Colposcopic assessment diagnosed ≤LSIL in 66 patients (56.9%) and HSIL in 50 patients (43.1%). Among the HSIL cases, lesion sizes ranged from 0.3 to 2.4 cm², with a mean size of 1.68 cm². Histological analysis confirmed ≤LSIL in 66 patients (56.9%) and HSIL in 50 patients (43.1%).

There were no statistically significant differences between the two groups in terms of age, parity, preoperative TCT results (ASC-US or higher), HPV infection type and frequency, colposcopic interpretation, transformation zone classification, or histologically confirmed HSIL rates (*P* > 0.05).

### 3.2 Comparison of surgical outcomes between the SCJ ablation and LEEP groups

Surgical outcomes for both groups are summarized here. The SCJ ablation group demonstrated a significantly shorter operative time compared with the LEEP group (7.46 ± 1.00 minutes vs. 13.15 ± 2.60 minutes, *P* < 0.05). Quantitative measurement of blood loss was not routinely recorded for all cases, and therefore is described qualitatively. Intraoperative blood loss in the SCJ ablation group was minimal and typically negligible, reflecting the non-excisional nature of the procedure. In contrast, patients in the LEEP group more frequently experienced measurable intraoperative bleeding requiring electrocautery hemostasis or gauze compression. In addition, SCJ ablation was associated with lower direct procedural costs compared with LEEP.

Margin status further distinguished the two groups (**Table 2**). As expected, no cases of positive surgical margins were identified in the SCJ ablation group, as no tissue excision was performed. In the LEEP group, 12 of 116 patients (10.3%) had positive surgical margins, including 10 cases with ectocervical margin involvement and 2 with endocervical margin involvement; no patients had positive deep (basal) margins.

**Table 2:** Comparison of postoperative margins, residual lesions, recurrence, and disease progression.

Category	SCJ ablation group (n=110)	LEEP group (n=116)	X²	*P*-value
Endocervical Margin Positive	0	2 (1.7%)	0.95	0.329
Ectocervical Margin Positive	0	10 (8.6%)	8.01	0.005
Deep Margin Positive	0 (0%)	0 (0%)		
Residual Lesion	0 (0%)	0 (0%)		
Postoperative Recurrence	0 (0%)	1 (0.86%)	0.95	0.329
Disease Progression	0 (0%)	0 (0%)		

Among the two patients with positive endocervical margins, one developed recurrent HSIL detected by endocervical curettage at the three-month follow-up visit. This patient subsequently underwent repeat LEEP, and no residual lesion was identified on histopathological evaluation at subsequent follow-up. No residual lesions or disease progression were observed in either group during the study period.

### 3.3 Comparison of postoperative complications and outcomes within one-year period between the SCJ ablation and LEEP groups

Postoperative complications and follow-up outcomes for both groups are presented in **Table 3**. In the LEEP group, 5 out of 116 patients (4.3%) required clinical intervention for excessive vaginal bleeding occurring between postoperative days 7 and 10. These patients also experienced a significantly longer duration of bleeding compared to those in the SCJ ablation group (*P* < 0.05). No cases of postoperative infection or cervical stenosis were reported in either group. The median wound healing time was shorter in the SCJ ablation group, averaging 4.5 weeks (range: 3–6 weeks), compared to 6.5 weeks (range: 5–8 weeks) in the LEEP group.

**Table 3:** Comparison of post-treatment outcomes within one year period.

Post-operative follow-up	Category	SCJ ablation group (n=110)	LEEP group (n=116)	X²	*P*-value
3 Months	≥ ASCUS	5 (4.5%)	3 (2.6%)	0.27	0.606
	hrHPV	40 (36.4%)	21 (18.1%)	7.57	0.006
	LSIL	0 (0%)	0 (0%)		
	HSIL	0 (0%)	1* (0.86%)	0.94	0.332
6 Months	≥ ASCUS	0 (0%)	0 (0%)		
	hrHPV	9 (8.2%)	11 (9.5%)	0.04	0.850
	LSIL	0 (0%)	0 (0%)		
	HSIL	0 (0%)	0 (0%)	0.00	0.970
9 Months	≥ ASCUS	0 (0%)	0 (0%)		
	hrHPV	0 (0%)	0 (0%)		
	LSIL	0 (0%)	0 (0%)		
	HSIL	0 (0%)	0 (0%)		
12 Months	≥ ASCUS	0 (0%)	5 (4.3%)	2.90	0.088
	hrHPV	0 (0%)	0 (0%)		
	LSIL	0 (0%)	6 (5.2%)	2.90	0.088
	HSIL	0 (0%)	0 (0%)		
18 Months	≥ ASCUS	8 (7.3%)	11 (9.5%)	0.26	0.613
	hrHPV	0 (0%)	0 (0%)		
	LSIL	10 (9.1%)	8 (6.9%)	0.15	0.696
	HSIL	0 (0%)	0 (0%)		

*This case had a positive endocervical resection margin.

At the three-month follow-up, cytological abnormalities (ASC-US or greater) were identified in 5 patients (4.5%) in the SCJ ablation group and 3 patients (2.6%) in the LEEP group, with no significant difference between groups (*P* = 0.606). No cytologic abnormalities were observed in either group at nine months. At 12 months, 5 LEEP patients (4.3%) exhibited cytologic abnormalities, while none were found in the SCJ ablation group. By 18 months, rates of ASC-US or greater were 7.3% (8/110) in the SCJ ablation group and 9.5% (11/116) in the LEEP group, with no significant difference.

High-risk HPV positivity was significantly higher in the SCJ ablation group at three months (36.4% vs. 18.1%, *P* = 0.006). However, by six months, hrHPV positivity had declined to 8.2% in the SCJ ablation group and 9.5% in the LEEP group, with no significant difference observed (*P* = 0.850).

No LSIL cases were detected in either group prior to 12 months. At the 12-month mark, LSIL was identified in 5 patients (5.2%) in the LEEP group, while none occurred in the SCJ ablation group. At 18 months, LSIL detection rates were 9.1% (10/110) in the SCJ ablation group and 6.9% (8/116) in the LEEP group; these differences were not statistically significant (*P* = 0.088; *P* = 0.696).

Only one HSIL recurrence was noted, occurring in a LEEP patient with a previously positive endocervical resection margin at the three-month follow-up. No additional HSIL cases were detected after six months, and no patients in either group progressed to squamous cell carcinoma during the follow-up period.

### 3.4 Comparison of outcomes beyond one-year period between the SCJ ablation and LEEP groups

Among patients with extended follow-up beyond one year, no cases of squamous cell carcinoma were detected in either group. The cumulative incidence of cytologic abnormalities (ASC-US or greater) from 18 months to the final follow-up was 5.2% (3/58) in the SCJ ablation group and 6.7% (4/60) in the LEEP group, with no statistically significant difference (*P* = 0.721).

Low-grade squamous intraepithelial lesions (LSIL) were identified in 2 SCJ ablation patients (3.4%) and 2 LEEP patients (3.3%) during extended follow-up (*P* = 0.658). All cases were managed conservatively without progression.

High-risk HPV persistence or re-detection beyond one year was observed in 3 patients (5.2%) in the SCJ ablation group and 4 patients (6.7%) in the LEEP group, with no significant difference (*P* = 0.731). Among these, most cleared the infection spontaneously during subsequent visits, and no associated HSIL recurrence was observed.

No additional HSIL recurrences occurred in either group beyond 12 months. Notably, all patients with initial positive surgical margins or abnormal cytology within the first year remained disease-free in the extended follow-up period.

Overall, the long-term outcomes of SCJ ablation were comparable to those of LEEP, with similar rates of cytologic abnormalities, LSIL, and HPV persistence, and no evidence of malignant progression in either group.

### 3.5 Comparison of CK7 expression before and after treatment in HSIL patients

A comparison of CK7 expression in pre- and post-treatment samples is presented in **Table 4**. Among the 226 patients with HSIL, a subset of 120 patients (60 from each treatment group) underwent CK7 immunohistochemical (IHC) staining. Paired cervical tissue samples, including endocervical curettage (ECC) specimens, were collected for each patient before and after treatment. Preoperative biopsies were obtained 2–5 weeks prior to intervention (mean: 3.5 weeks), and postoperative samples were collected during the three-month follow-up visit.

**Table 4:** Correlation between CK7+ SCJ cell changes and treatment outcomes.

Category	SCJ group(n=60)		LEEP group(n=60)		X^2^	*P*-value
	Cases N	Percentage (%)	Cases N	Percentage (%)		
Pre-treatment CK7 Expression					0.29	0.592
Negative	12	20.0	15	25.0		
Positive	48^*^	80.0	45	75.0		
Post-treatment CK7 Expression					0.39	0.531
Negative	52	86.7	50	83.3		
Positive	5^**^	13.3	10	16.7		
X^2^	36.66		26.60			
*P* Value	<0.001		<0.001			

*Most CK7-positive cases were HSIL, with a few SCJ cells present.

**These CK7-positive cases represented squamous epithelial metaplasia cells, with no SCJ cells detected. See results section for details.

Two distinct types of CK7-positive non-columnar epithelial cells were observed in pre-treatment specimens: dysplastic HSIL cells and non-dysplastic squamous epithelial cells originating from the squamocolumnar junction (SCJ cells). In the SCJ ablation group, 48 patients exhibited CK7-positive staining, while 12 showed no expression. Among the 48 positive cases, 16 contained only HSIL cells, 27 had both HSIL and SCJ cells, and 5 demonstrated only SCJ cells. In the LEEP group, 45 cases were CK7-positive and 15 were negative. Of the positive cases, 15 had only HSIL, 27 had both HSIL and SCJ cells, and 3 showed SCJ cells alone.

**Figure 2:**
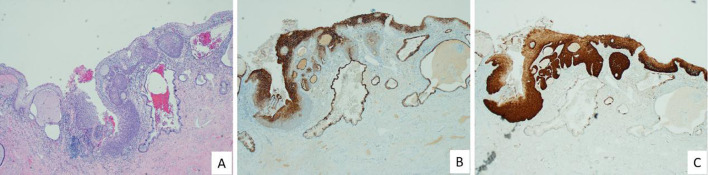
High-grade squamous intraepithelial lesion (HSIL) in the transformation zone with CK7 and p16 expression prior to SCJ ablation or LEEP treatment. **(A)** A cervical intraepithelial neoplasia grade 2 (CIN2) lesion is evident within the transformation zone. **(B)** CK7 immunostaining highlights the lesion, showing strong cytoplasmic staining within the transformation zone. CK7-positive endocervical glandular epithelium serves as an internal positive control. **(C)** Diffuse “block” positivity for p16 confirms the presence of HSIL.

**Figure 3:**
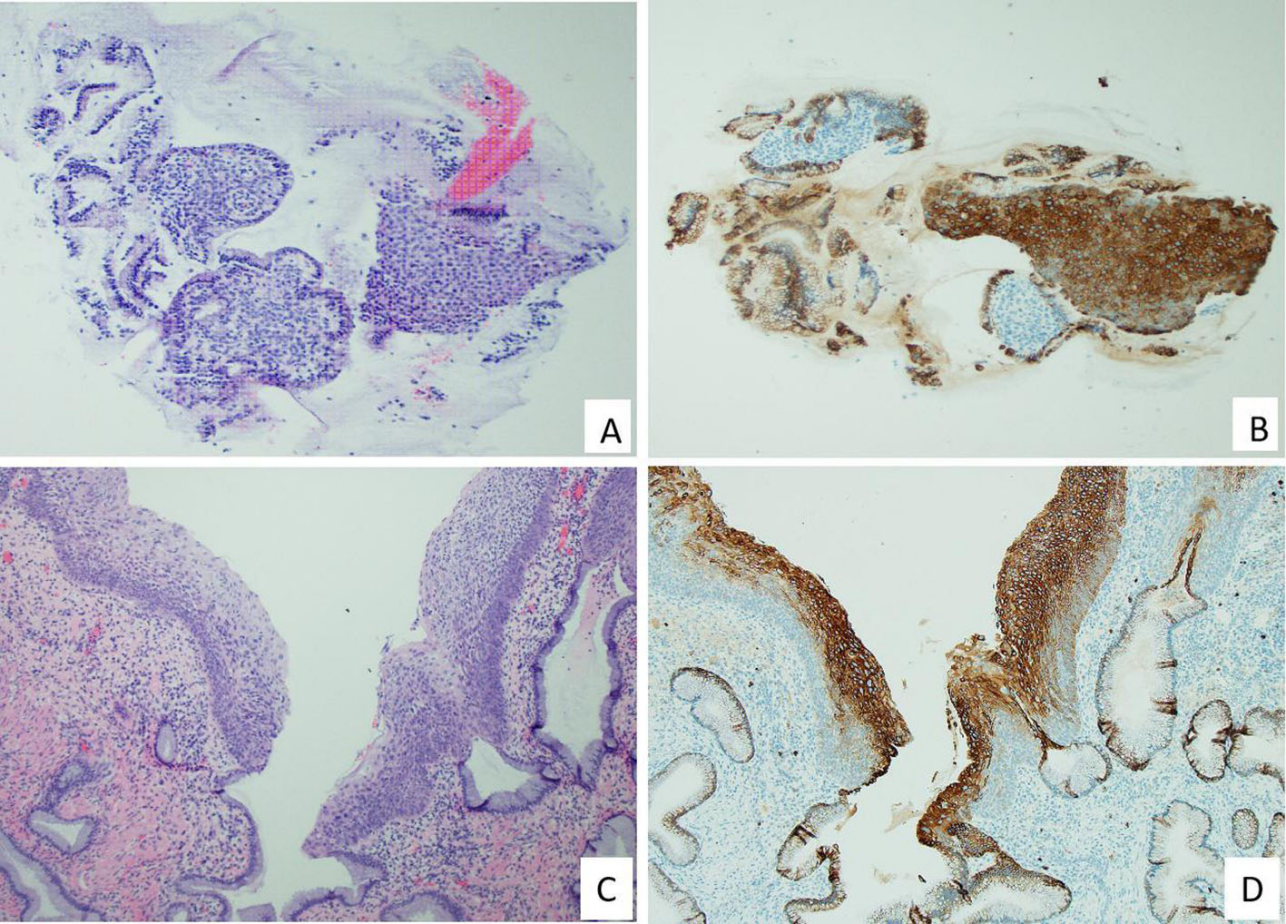
Endocervical curettage and cervical biopsy findings three months after thermal ablation. A 35-year-old patient underwent SCJ-targeted thermal ablation and presented for follow-up with a Pap smear showing ASC-US and positive high-risk HPV. Colposcopic examination revealed no visible abnormalities. Endocervical curettage and cervical biopsy demonstrated benign endocervical tissue with metaplastic squamous epithelium **(A)**, and chronic cervicitis with squamous metaplasia in the SCJ region **(C)**. CK7 immunostaining revealed positive cytoplasmic staining in metaplastic squamous epithelial cells **(B, D)** and endocervical glandular cells **(B, D)**. No evidence of squamous intraepithelial lesion (SIL) was identified.

Post-treatment biopsy specimens revealed only CK7-positive squamous metaplastic cells, with no residual CK7-positive HSIL or SCJ cells detected in either group. CK7-positive metaplastic cells were observed in 5 patients from the SCJ ablation group and in 10 patients from the LEEP group. Representative images of CK7 immunostaining patterns in pre- and post-treatment tissues are displayed in **Figures 2**, **3**.

## 4 Discussion

Before addressing the unique advantages of cervical SCJ-targeted ablation, it is important to contextualize its role by reviewing the strengths and limitations of loop electrosurgical excision procedure (LEEP), which remains the most widely used intervention for cervical precancerous lesions. Successful LEEP treatment depends on comprehensive colposcopic assessment of the transformation zone (TZ), including lesion extent, endocervical involvement, and visibility of the squamocolumnar junction (SCJ) (5, 6, 10). As both a diagnostic and therapeutic modality, LEEP effectively reduces the risk of progression to invasive carcinoma. However, residual or recurrent disease still occurs, with reported rates of residual disease ranging from 3.2% to 3.6%, recurrence within two years of approximately 1.3%, and progression to invasive cancer at approximately 0.3% (11). Identified risk factors include older age, positive surgical margins—particularly endocervical—and abnormal cytology during follow-up (11).

A further concern with LEEP is its impact on reproductive outcomes. Excisional procedures shorten cervical length and may impair its structural integrity, increasing the risk of cervical insufficiency, spontaneous preterm birth, and miscarriage (5, 6, 12, 13). The extent of excision correlates with obstetric risk: deeper resections are associated with higher rates of preterm birth and adverse neonatal outcomes. Large population-based studies have demonstrated a dose–response relationship between excision depth and preterm delivery risk (14). More recent evidence continues to support these associations and highlights the importance of minimizing cervical trauma when clinically appropriate (8).

Given the increasing incidence of cervical intraepithelial lesions in women of reproductive age, there is a growing need to balance oncologic efficacy with preservation of cervical function. In carefully selected patients—particularly those with visible TZ (types 1 and 2) and limited lesion extent—minimally invasive approaches may offer a reasonable alternative to excisional treatment. In the present study, LEEP excision depth ranged from 1.0–1.5 cm, with ectocervical margin positivity in 8.6% and endocervical margin positivity in 0.86% of cases. One patient with a positive endocervical margin developed recurrent HSIL requiring repeat excision, while the remaining patients remained disease-free during follow-up.

Margin status is traditionally regarded as an important predictor of residual or recurrent disease; however, its prognostic significance is increasingly recognized as context-dependent. Recent studies have suggested that positive margins do not uniformly predict recurrence and that conservative follow-up may be appropriate in selected patients when close surveillance is feasible (15). These findings are consistent with our observations and support a more individualized approach to post-LEEP management. In this context, HPV persistence has emerged as an important complementary risk factor. Integration of HPV testing, and potentially HPV genotyping, may enhance risk stratification beyond margin status alone and improve long-term surveillance strategies.

The SCJ represents a key histological and biological site for the development of cervical squamous neoplasia. It is important to distinguish the SCJ region from SCJ cells, a specialized epithelial population with stem-like properties that are highly susceptible to high-risk HPV infection (4). More than 90% of HSIL cases are believed to arise from these cells, which exhibit a distinct immunophenotype and contribute to the early stages of carcinogenesis (4, 7, 16–18). Importantly, SCJ cells do not regenerate following removal, supporting the concept that targeted elimination of these cells may reduce the risk of recurrence. This biological framework provides the rationale for SCJ-targeted ablation.

Despite this mechanistic rationale, clinical studies specifically evaluating SCJ-targeted ablation remain limited. Most prior work has focused on laser-based ablation, which has shown variable outcomes, including relatively high recurrence rates and challenges in achieving consistent treatment depth (19–21). Similarly, data on electrocautery-based thermal ablation are still emerging (22). Existing studies suggest that thermocoagulation is comparable to cryotherapy in safety and efficacy, with advantages including shorter procedure time, minimal bleeding, and lower cost (22). In our cohort, no severe procedural complications or significant discomfort were observed, supporting the feasibility of this approach.

Compared with LEEP, SCJ-targeted ablation demonstrated several procedural advantages, including shorter operative time, minimal intraoperative blood loss, lower cost, and fewer postoperative complications. These features may be particularly relevant in low-resource settings and in patients seeking to minimize cervical trauma. Follow-up data showed low rates of cytological abnormalities, HPV persistence, and lesion recurrence in both groups, with no progression to invasive carcinoma observed. Although hrHPV positivity was higher in the ablation group at three months, this difference resolved over time, suggesting that early post-treatment HPV detection may not necessarily indicate treatment failure.

Importantly, given the non-randomized design and low event rates, these findings should be interpreted cautiously. The present study does not establish equivalence between SCJ ablation and LEEP but rather suggests that SCJ-targeted ablation may achieve similar short- to mid-term clinical outcomes in carefully selected patients with visible transformation zones.

A key innovation of this study is the application of CK7 immunohistochemistry to evaluate SCJ cell dynamics. CK7-positive SCJ cells have previously been associated with increased risk of progression in low-grade lesions (23, 24). In our study, both SCJ ablation and LEEP were associated with a marked reduction in CK7-positive SCJ cells, with no residual CK7-positive HSIL or SCJ cells identified in post-treatment samples. These findings support the concept that SCJ cells represent a biologically relevant therapeutic target. However, as CK7 analysis was performed in a subset of patients and not directly linked to long-term clinical outcomes, these findings should be considered exploratory.

Despite these encouraging findings, several important limitations must be acknowledged. First, the study was not randomized, and treatment allocation was influenced by patient preference and clinical judgment, introducing potential selection bias. Second, the low number of recurrence events limits statistical power and precludes definitive comparative conclusions. Third, long-term follow-up was available only for a subset of patients. In addition, the absence of post-treatment histopathologic specimens in the ablation group is an inherent limitation of non-excisional approaches. Finally, reproductive outcomes were not assessed, and therefore the potential impact of SCJ ablation on fertility and pregnancy outcomes remains to be determined.

In summary, in this prospective observational cohort, SCJ-targeted thermal ablation demonstrated favorable procedural characteristics and similar observed short- to mid-term clinical outcomes compared with LEEP in carefully selected patients. The observed reduction in CK7-positive SCJ cells provides additional biologic insight into SCJ-targeted therapy, although these findings remain exploratory. Further studies incorporating randomized designs, HPV genotyping, and long-term clinical and reproductive outcomes are needed to better define the role of SCJ-targeted ablation in the management of cervical intraepithelial lesions.

## Data Availability

The original contributions presented in the study are included in the article/supplementary material. Further inquiries can be directed to the corresponding authors.

## Glossary

SCJ: Squamous Columnar Junction
CIN: Cervical Intraepithelial Neoplasia
HSIL: High-grade Squamous Intraepithelial Lesions
LSIL: Low-grade Squamous Intraepithelial Lesions
LEEP: Loop Electrosurgical Excision Procedure
TZ: Transformation Zone
hrHPV: High-risk Human Papillomavirus
SIL: Squamous Intraepithelial Lesions
ECC: Endocervical Curettage
